# Supplementary material for: Complexome profiling of the Chlamydomonas psb28 mutant reveals TEF5 as an early PSII assembly factor
Source: Plant Cell. 2025 Mar 17;37(6):koaf055. doi: 10.1093/plcell/koaf055 (PMC12164590; doi:10.1093/plcell/koaf055)
Supplement: koaf055_Supplementary_Data [file koaf055_supplementary_data.zip › psb28_interactive.zip!/plots/intensity/qualityPlots/peptides/NUOA9_Cre10.g434450.t1.2.html]

### Cre10.g434450.t1.2

Trivial name: NUOA9  
SeqCoverage [median]: 49.60  
Mass: 43.65 kDa  
  
 

| Sequence | MissedCleavage | Mass [Da] | wtIntensity | muIntensity | Score |
| --- | --- | --- | --- | --- | --- |
| AMYKPFDSVR | 1 | 1212.60 | 22387.49 | 10384.49 | 94.62 |
| DDTVHLPAWAVK | 0 | 1350.69 | 449.88 | 0.00 | 91.55 |
| DIPESVR | 0 | 814.42 | 545.40 | 0.00 | 64.78 |
| DKVVPAGALGYADLGIVPQK | 1 | 2010.12 | 1284.51 | 919.60 | 80.16 |
| EVLDAFPDATIVR | 0 | 1444.76 | 601.74 | 0.00 | 95.57 |
| EVYDLLLK | 0 | 991.56 | 26444.50 | 2493.16 | 125.25 |
| IYRDDTVHLPAWAVK | 1 | 1782.94 | 11653.00 | 18740.32 | 100.11 |
| LAAETGQVQR | 0 | 1071.57 | 55784.94 | 53113.99 | 226.04 |
| LIHFSDMGADENHK | 0 | 1612.73 | 29068.10 | 22638.98 | 133.15 |
| MLPGLPMTSPLATEDYVEEMLR | 0 | 2492.20 | 1232.68 | 0.00 | 44.10 |
| MLPGLPMTSPLATEDYVEEMLRDK | 1 | 2735.32 | 2576.00 | 0.00 | 53.44 |
| NDDDIKR | 1 | 874.41 | 1335.44 | 749.32 | 140.98 |
| NWSFEDVHVDFPK | 0 | 1618.74 | 5757.81 | 0.00 | 95.40 |
| NWSFEDVHVDFPKR | 1 | 1774.84 | 2524.49 | 0.00 | 69.72 |
| QMGDLGQIVLLPELDIR | 0 | 1909.03 | 1924.42 | 0.00 | 93.75 |
| QMGDLGQIVLLPELDIRNDDDIKR | 2 | 2765.44 | 2441.91 | 0.00 | 64.87 |
| STENEAMHLK | 0 | 1158.53 | 20841.88 | 18137.75 | 103.70 |
| TLYLGGPEVLTMR | 0 | 1448.77 | 14864.29 | 15297.67 | 111.86 |
| VTDGLAIEPVR | 0 | 1168.65 | 32762.89 | 38930.54 | 205.59 |
| VVPAGALGYADLGIVPQK | 0 | 1766.99 | 28128.82 | 0.00 | 127.02 |
| YYNIKQ | 1 | 827.42 | 5462.67 | 7137.06 | 148.12 |
|  |  |  | 268072.9 | 188542.9 |  |
